# Significance of low-dose radiation distribution in development of radiation pneumonitis after helical-tomotherapy-based hypofractionated radiotherapy for pulmonary metastases

**DOI:** 10.1093/jrr/rrt080

**Published:** 2013-06-11

**Authors:** In-Young Jo, Chul-Seung Kay, Ji-Yoon Kim, Seok-Hyun Son, Yong-Nam Kang, Ji-Young Jung, Ki-Jun Kim

**Affiliations:** 1 Department of Radiation Oncology, The Catholic University of Korea College of Medicine, 222 Banpodaero, Seochogu, Seoul, 137-701, South Korea; 2 Department of Diagnostic Radiology, The Catholic University of Korea College of Medicine, 222 Banpodaero, Seochogu, Seoul, 137-701, South Korea

**Keywords:** low-dose radiation distribution, radiation pneumonitis, hypofractionated radiotherapy, helical tomotherapy, pulmonary metastases

## Abstract

Hypofractionated radiotherapy (HRT) is now commonly used for pulmonary malignancies, since a tumoricidal dose can be accurately delivered to the target without a consequential dose to adjacent normal tissues. However, radiation pneumonitis (RP) is still a major problem after HRT. To determine the significant parameters associated with developing RP, we retrospectively investigated data from patients with lung metastases treated with HRT using helical tomotherapy. A total of 45 patients were included in the study and the median age was 53 years old. The median prescriptive doses were 50 Gy to the internal target volume and 40 Gy to the planning target volume in 10 fractions over 2 weeks. RP was diagnosed by chest X-ray or computed tomography after HRT, and its severity was determined by CTCAE version 4.0. The incidence of symptomatic RP was 26.6%. Univariate analysis indicated that mean lung doses, V5, V10, V15, V20 and V25 were associated with the development of symptomatic RP (*P* < 0.05). However, multivariate analysis indicated that only V5 was associated with the development of symptomatic RP (*P* = 0.019). From the ROC curve, V5 was the most powerful predictor of symptomatic RP, and its AUC (area under curve) was 0.780 (*P =* 0.004). In addition, the threshold value of V5 for the development of symptomatic RP was 65%. A large distribution of low-dose radiation resulted in a higher risk of lung toxicity. So, to prevent symptomatic RP, it is recommended that the V5 be limited to <65%, in addition to considering conventional dosimetric factors. However, further clinical study must be undertaken in order to confirm this result.

## INTRODUCTION

Hypofractionated radiotherapy has been increasingly used to treat patients with pulmonary malignancies, including metastases, because this technology makes it possible to accurately deliver a highly ablative dose of radiation to the target, without a consequential dose to adjacent lung tissues. The local control rate for early-stage non-small-cell lung cancer can reach 90–100% within a 3–5 year follow-up period [1, 2]. Okunieff *et al.* reported an excellent local control rate of 88% in patients treated with stereotactic body radiotherapy (SBRT) for multiple lung metastases [3], and we also reported successful use of hypofractionated radiotherapy using helical tomotherapy for multiple lung metastases [4]. However, the risk estimation of a pulmonary toxicity after such treatment was not well understood at that time. Radiation pneumonitis (RP) is a common repercussion of such treatments and can sometimes be fatal, despite further evolution of radiotherapy techniques. Therefore, the prevention of RP, believed possible through thorough analysis of pulmonary dosimetric parameters, is highly desirable.

A variety of authors from different institutes have reported on the treatment of patients with pulmonary malignancies, including metastases, using a range of radiation techniques and dose schedules. Patients with varying characteristics, including the size and number of pulmonary nodules and the status of pulmonary function, have been included in their studies. Furthermore, differences also existed in the aims of the radiotherapy and in the experience of the radiation oncologist [1–5]. Such multiplicities of factors in treatments, patients and physicians make it difficult to construct prediction models for RP. We retrospectively investigated the pulmonary dosimetric parameters in order to investigate their relationship to symptomatic RP after hypofractionated radiotherapy for pulmonary metastases.

## MATERIALS AND METHODS

We performed this study after approval by the Catholic University of Korea Incheon St Mary Hospital Institutional Review Board. The medical records, including radiation dosimetry, treatment plans and sequential radiologic films, were reviewed. The eligibility criteria in this study were age >18 years, an ECOG (Eastern Cooperative Oncology Group) performance status ≤ 2, a completely controlled primary tumor, definitive lung nodules or masses in computed tomography, at least 1200 ml normal lung volume which was not associated with the planning target volume (PTV), a favorable pulmonary status consisting of a forced expiratory volume for 1 s (FEV1) and a diffusion capacity of carbon monoxide (DLCO) > 40% of the prediction by pulmonary function test, ≥ 3000 per microliter white blood cells, ≥ 70 000 per microliter platelets, and recurrent or metastatic tumors after previous treatment including surgery, radiotherapy or systemic chemotherapy. In patients with multiple lung metastases, their disease status was required to be at least stable after previous systemic chemotherapy. Patients were excluded from this study if progressive disease had been noted after the previous systemic treatment.

Of the 52 patients treated with hypofractionated radiotherapy for pulmonary malignancies (including metastases), 45 patients who met these criteria were identified between November 2005 and September 2009. We have previously made a detailed description of our treatment protocol in a previous report [4], and it is briefly described here.

After training the patients to breathe shallowly, we immobilized them for an initial simulation and repeated the treatment using the BodyFix system (Medical Intelligence, GmBH, Schwabmunchen, Germany), using a low-pressure foil for their abdominal dampening. We then obtained two series of CT scans for shallow breathing, one series in the inspiratory and the other in the expiratory phase, in order to track the physiologic respiratory motion of the tumors and internal organs. An internal target volume (ITV) was defined through the superimposition of each visible tumor on each series of CT scans. The margin of the PTV was 1 cm beyond the ITV; the adjacent normal structures, such as normal lung tissues, esophagus, spinal cord, liver and heart, were contoured in each series of CT scans. Treatment planning was then performed using Tomotherapy Planning Station (TomoTherapy, Madison, WI). The treatment aims were curative for ≤5 metastases and palliative for >5 metastases.

The median doses prescribed were 50 Gy and 40 Gy delivered in 10 fractions over 2 weeks to the 95% isodose volume to the ITV and PTV, respectively. While achieving the target dose, the dose constraints for each specific organ at risk (OAR) were kept below a maximum of 30 Gy and a maximum of 40 Gy to the spinal cord and esophagus, respectively, and the constraint dose to the liver was two-thirds of the normal liver receiving < 30 Gy, and at least 700 ml of normal liver tissue not included in the PTV. We tried to minimize the risk of pulmonary toxicity by decreasing the mean lung dose (MLD) and by keeping the percentage of lung volume receiving > 25 Gy (V25) as low as possible, so that 60% of the normal lung received < 2 Gy per fraction, and at least 1200 ml of normal lung was not included in the PTV [3]. Before each treatment, we performed megavoltage CT (MVCT) scanning and registration of images from simulation CT and MVCT in three axes (*x*-, *y*-, *z*-axis) and rotation. After confirmation of appropriate coverage of the ITV and the PTV, we treated the patients with prescriptive isodose lines.

After completion of a treatment, we observed the patients two weeks after the treatment, and every three months thereafter. An RP was diagnosed by serial follow-up interviews and radiologic studies, including chest X-ray and CT scans, and through discussion with the pulmonologist, diagnostic radiologist and radiation oncologist of each patient. The grade of pneumonitis was defined through the Common Terminology Criteria for Adverse Events, version 4.0 (CTCAE 4.0): Grade 1 (asymptomatic or requiring no treatment), Grade 2 [symptomatic, requiring medical intervention or limiting instrumental active daily life (ADL)], Grade 3 (severely symptomatic, limiting self care and ADL, or oxygen indicated), Grade 4 ([life-threatening, respiratory compromise, urgent intervention indicated (e.g. tracheostomy or intubation)], and Grade 5 (death). The pulmonary dosimetric parameters, [including MLD and lung volume receiving at least n Gy of radiation (Vn)], were extracted from the dose–volume histogram (DVH). The primary endpoint of this study was the development and severity of RP, and the secondary was the development of ≥ Grade 2 RP. A cross tabulation, an independent *t*-test, a multiple logistic regression and a receiver–observer curve (ROC) were used to analyze the data.

## RESULTS

The follow-up period was from 1–38 months, with 12 months being the median value. RP developed in 37 of the total 45 patients (82.2%); 25 (55.6%) had Grade 1 RP, 9 (20.0%) had Grade 2 RP, and 3 (6.6%) had Grade 3 RP. Grade 4 or greater RP was not apparent in any of the patients. The median interval between the completion of radiotherapy and the development of RP was 3 months (range, 1–12 months). Table 1 shows the characteristics of the patients and the breakdown of RP incidence. No significant correlation was found between the incidence of Grade 2–3 RP and clinical parameters, such as age, sex, ECOG performance status, number of lesions, previous history of radiotherapy or chemotherapy, sums of PTVs, daily dose, total dose or pulmonary function status (including FVC, FEV1 and DLCO).

**Table 1.** Patient characteristics (*n* = 45)

		Number of patients (%)	Incidence of RP (%)		Statistical significance
			Grade 0–1	Grade 2–3	
**Age (years)**	Median 53		Median 52.5	Median 59.0	ns
	Range 33–81				
**Sex**	Male	24 (53.3)	19 (42.2)	5 (11.1)	ns
	Female	21 (46.7)	14 (31.1)	7 (15.6)	
**ECOG**	0, 1	37 (82.2)	30 (66.7)	7 (15.6)	ns
	2	8 (17.8)	3 (6.6)	5 (11.1)	
**Previous RT**	yes	5 (11.1)	5 (11.1)	0	ns
	no	40 (88.9)	28 (62.2)	12 (26.7)	
**Previous CTx**	yes	20 (44.4)	14 (31.1)	6 (13.35)	ns
	no	25 (55.6)	19 (42.2)	6 (13.35)	
**Number**	≤5	31 (75.6)	21 (46.6)	8 (17.8)	ns
**of lesions**	>5	14 (24.4)	12 (26.7)	4 (8.9)	
**Sum of PTV**	≤200 ml	23 (53.3)	19 (42.2)	4 (8.9)	ns
**volume**	>200 ml	22 (46.7)	14 (31.1)	8 (17.8)	
**Daily/Total dose**	<5.0/50	19 (42.2)	14 (31.1)	5 (11.1)	ns
**(Gy)**	≥5.0/50	26 (57.8)	19 (42.2)	7 (15.6)	
**PFT FVC (% of predictive value)**			Median 93.1	Median 114.0	ns
**FEV1 (% of predictive value)**			Median 89.7	Median 106.3	
**DLCO (% of predictive value)**			Median 67.6	Median 68.0	

RP = radiation pneumonitis, ECOG = Eastern Cooperative Oncology Group, RT = radiotherapy, CTx = chemotherapy, PTV = planning target volume, PFT = pulmonary function test, FVC = forced vital capacity, FEV1 = forced expiratory volume for 1 s, DLCO = diffusion capacity of carbon monoxide.

**Fig. 1.**
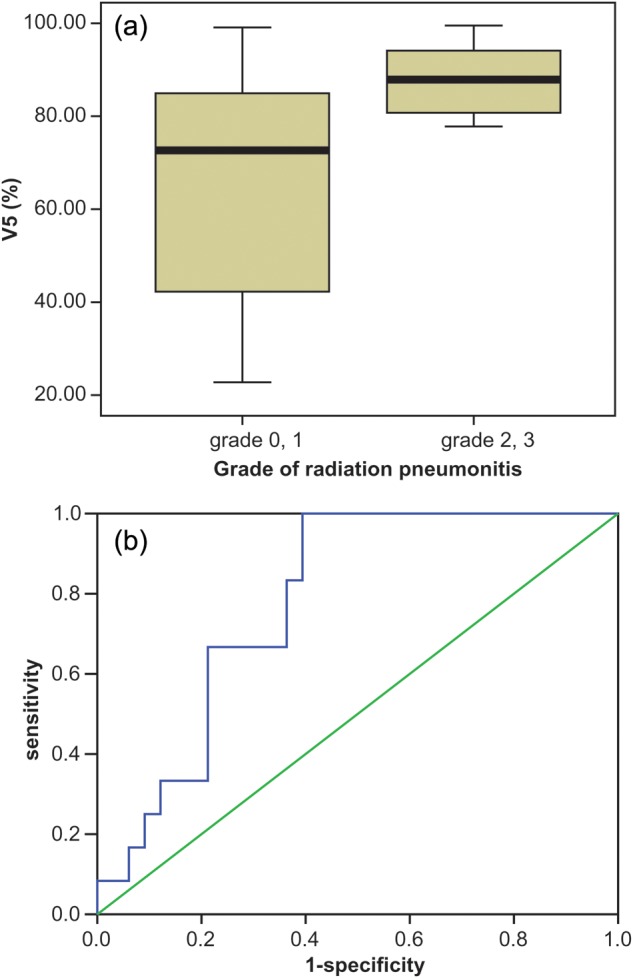
The box plot and ROC curve of V5 relative to the development of Grade 0–1 and Grade 2–3 RP. (**a**) The box plot shows that the values of the mean ± standard deviation for V5 were (65.65 ± 24.02)% and (87.73 ± 7.47)% for Grade 0–1 and Grade 2–3 RP, respectively. This difference was statistically significant in both univariate (*P* = 0.003) and multivariate analysis (*P* = 0.019). (**b**) The ROC curve of V5 yielded 0.780 of the AUC (area under curve), and this was statistically significant (*P* = 0.004). V5 was the most powerful predictor of the pulmonary dosimetric parameters, including mean lung dose and Vn (volume of percentage of tumor-free normal lung receiving ≥n Gy).

**Fig. 2.**
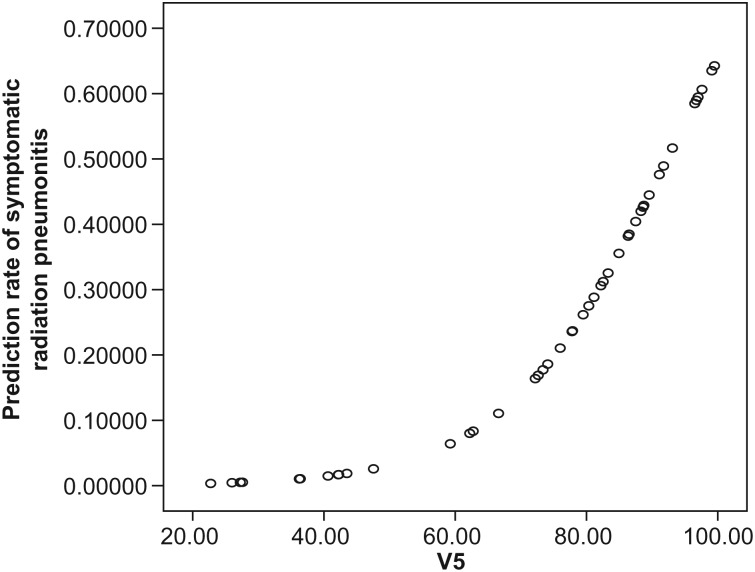
Predicted rate of symptomatic RP vs V5. The predicted rate of symptomatic RP increase was related to increase in V5, and rose abruptly above 65% V5. Thus, this could be considered the ‘threshold value’ in development of Grade 2–3 symptomatic RP.

Univariate analysis showed a statistically significant association (*P* < 0.05) between the development of Grade 2–3 RP and pulmonary dosimetric parameters, including V5, V10, V15, V20, V25 and MLD. The ROC curve indicated similar outcomes to those of the univariate analysis, and the AUC (area under curve) value was highest for V5 (AUC = 0.780, *P =* 0.004). However, in multivariate analysis, V5 was the only statistically significant parameter associated with Grade 2–3 RP (Table 2). Figure 1 shows (**a**) a comparison of the mean and standard deviation of V5 for Grade 0–1 and Grade 2–3 RP through box plots from independent *t*-tests, and (**b**) the sensitivity vs one minus specificity of V5 of developing Grade 2–3 RP with a ROC curve. Figure 2 shows the curve of predicted rate for developing Grade 2–3 RP. The prediction curve increases with increase in V5, and then bends sharply up at ∼ 65%. This suggests that the risk of Grade 2–3 RP may abruptly increase when V5 is greater than 65%, and that this is the threshold value for limiting risk of developing Grade 2–3 RP to < 10%.

**Table 2.** Analysis of relationship between pulmonary dosimetric parameters and development of ≥Grade two radiation pneumonitis

		Mean	SD	Univariate	ROC		Multivariate
					AUC	*P*-value	
V5	Grade 0–1	65.65	24.02	0.003	0.780	0.004	0.019
	Grade 2–3	87.75	7.47				
V10	Grade 0–1	46.58	25.07	0.026	0.707	0.035	0.164
	Grade 2–3	64.42	15.50				
V15	Grade 0–1	29.12	16.60	0.026	0.710	0.033	0.674
	Grade 2–3	41.45	13.33				
V20	Grade 0–1	18.06	9.43	0.027	0.682	0.065	0.674
	Grade 2–3	25.69	11.11				
V25	Grade 0–1	12.12	6.87	0.015	0.707	0.035	0.579
	Grade 2–3	17.51	7.21				
V30	Grade 0–1	8.02	5.28	0.083	0.734	0.018	
	Grade 2–3	12.74	6.13				
V40	Grade 0–1	3.71	3.36	0.364	0.595	0.336	
	Grade 2–3	4.82	4.23				
V50	Grade 0–1	0.55	0.70	0.128	0.563	0.572	
	Grade 2–3	1.01	1.22				
MLD	Grade 0–1	11.53	4.67	0.006	0.758	0.009	0.512
	Grade 2–3	15.71	2.88				
NTCP	Grade 0–1	2.99	5.88	0.230	0.673	0.091	
	Grade 2–3	6.30	11.66				

SD = standard deviation, ROC = receiver observer curve, AUC = area under curve, V5–25 = volume percentage of tumor-free normal lung receiving at least 5–25Gy, MLD = mean lung dose, NTCP = normal tissue complication probability.

## DISCUSSION

The recent evolution of radiation therapy, such as SBRT or hypofractionated radiotherapy, has been accompanied by advancements in image guidance and intensity modulation. Image guidance through fluoroscopy or computed tomography enables a more accurate delivery of radiation to the target, and intensity modulation makes it possible to delicately modulate radiation doses between the target and the adjacent normal tissues [6]. However, intensity-modulated radiotherapy, while endeavouring to achieve better conformity around the target, cannot avoid a large distribution of low-dose radiation, and the clinical significance of a large area of low-dose radiation has not been investigated in any depth. Nowadays, to achieve better conformal dose distribution around the target, most physicians are using a greater number of radiation fields for the SBRT technique, non-coplanar conformal radiotherapy, intensity-modulated radiotherapy, and dynamic conformal multiple-arc radiotherapy, thus the low-dose exposure area of normal tissue is inevitably increased [5].

**Fig. 3.**
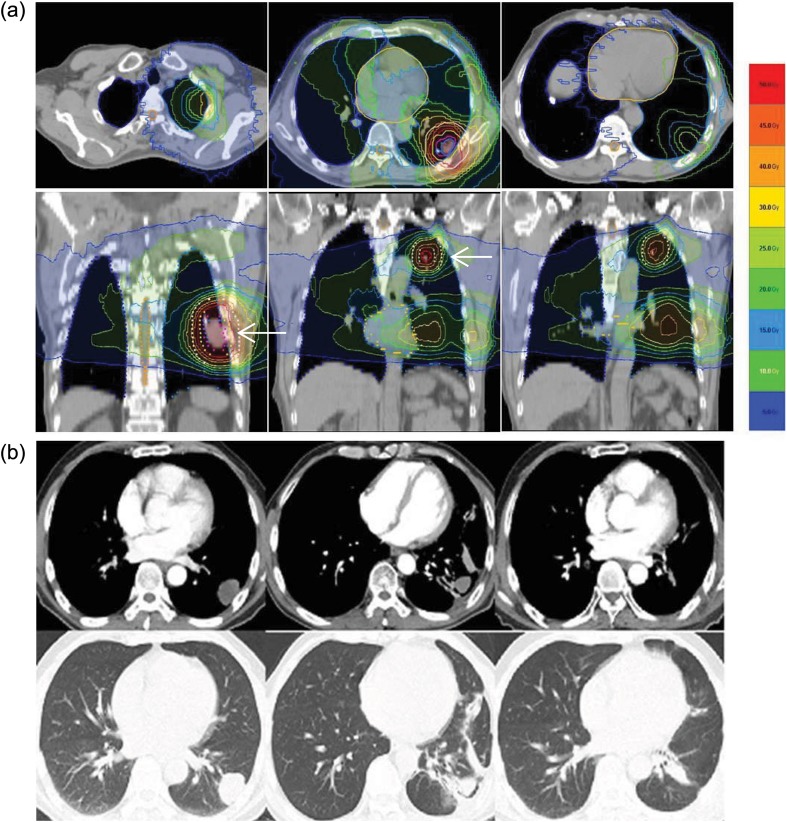
Case presentation: a 60-year-old man with multiple lung metastases (×4) from hard palate cancer. The primary site was surgically removed five years ago. After detection of multiple lung metastases, the patient was treated with systemic chemotherapy and the response was stable. Treatment with hypofractionated radiotherapy using helical tomotherapy was decided upon. The prescriptive dose was 50 Gy to the ITV (internal target volume) and 40 Gy to the PTV (planning target volume) in 10 fractions over 2 weeks. (**a**) The treatment plan with isodose lines superimposed on the planning computed tomography. The following isodose lines are shown: 50 Gy, 45 Gy, 40 Gy, 30 Gy, 25 Gy, 20 Gy, 15 Gy, 10 Gy and 5 Gy. The PTV of two metastatic nodules in the left lung was surrounded by the 40 Gy line white arrows. However, the 10% isodose line (5 Gy) surrounded almost the entire lung area. The mean lung dose and V5 for this patient were 14.98 Gy and 88%, respectively. (**b**) The computed tomography before radiotherapy showed a metastatic nodule in the left lower lung, and the total number of lung metastases was four (not shown) (two images on the left). The computed tomography three months after radiotherapy showed radiation pneumonitis. The patient complained of a dry cough but did not require medication, so we categorized this as Grade 2 RP. The morphologic changes due to RP, as seen in the CT scan, were limited to V30, a small volume of the lung receiving a relatively high dose, therefore, symptoms such as the dry cough might have been caused by the large distribution of the low dose, for example V5 (central two images). One year after completion of radiotherapy, the computed tomography showed minimal radiation fibrosis, and the patient did not complain of respiratory-related symptoms (two images on the right).

One case history has been presented to show the relatively large low-dose distribution and the development of Grade 2 RP (Figure 3). Helical tomotherapy, which continuously delivers radiation to the target, rotating 360° around the patient [7], is an extreme example of this, so the effect of a relatively large distribution of low-dose radiation is of particular concern with this therapy.

In the case of radiation therapy for pulmonary malignancies, both the MLD, which increases according to the increase in total dose to the tumor, and also the dose–volume relationship, i.e. the volume percentage receiving n Gy of radiation (Vn), are thought to be very important factors contributing to the development of RP. Graham *et al.* reported V20 as the best predictor of ≥ Grade 2 RP after 3D conformal radiotherapy with a conventional fractionation schedule [8]. V20 in conventional radiotherapy can be approximately translated to V16 in hypofractionated radiotherapy, with 50 Gy delivered in 5 Gy per fraction, using the linear quadratic model with an α/β ratio of 3.3 ± 1.5 Gy [9]. However, in the field of SBRT for pulmonary malignancies, despite many investigators’ efforts, the relationship between the dose–volume parameter and symptomatic RP, or the parameter predicting the risk of symptomatic RP has not been reported in depth.

There is debate as to whether ‘a large dose to a small area’ or ‘a small dose to a large area’ is more likely to result in RP [10]. Borst *et al.* reported that the incidences of RP were 10.9% and 17.6% for SBRT and conventional fractionated radiotherapy (CFRT) patients, respectively. They found a significant dose–response relationship between RP and MLD after SBRT, and this outcome was similar to that of RP after CFRT [11]. However, Guckenberger *et al.* reported the association of low-dose radiation distribution with the development of RP after SBRT, with an incidence of RP of 18.6%. The MLD was 12.5 ± 4.3 Gy and 9.9 ± 5.8 Gy for patients with and without the development of RP, respectively, and this group suggested a low-dose radiation parameter of V2.5 as the best-fitting value for the development of RP in log likelihood values [12]. This low-dose radiation association with the development of RP has been supported by previous conventional treatment data. Gopal *et al.* reported a decrease of diffusion capacity after definitive radiation therapy in lung cancer patients (*n* = 26). In their report, V13 was suggested as a threshold dose for deterioration of diffusion capacity after radiotherapy [13].

Yamashita *et al.* reported that the incidence of lung toxicity increased when a large volume of lung parenchyma was irradiated to doses as high as the minimum dose to the PTV, even with SBRT [14]. In a series of helical tomotherapy, Song *et al.* treated 37 patients with non-small-cell lung cancer with concurrent chemoradiotherapy, using helical tomotherapy, up to a total dose of 60–70.4 Gy at 2.4 Gy per fraction to the GTV, and 50–60 Gy at 1.8–2.0 Gy per fraction to the PTV. The incidence of RP ≥ Grade 3 was 18.9% and the V5 of the contralateral lung was the only significant predictor of RP ≥Grade 3 in multivariate analysis. They suggested that the V5 of the contralateral lung should be kept as low as possible (<60%), in addition to the conventional dosimetric factors [15].

In univariate analysis of our data, MLD and Vn (n = 5, 10, 15, 20, 25) were related to the development of symptomatic RP with statistical significance. However, in multivariate analysis, MLD was not significant, and V5 was the only statistically significant factor associated with development of symptomatic RP. This means that V5 was the most powerfully significant parameter to predict ≥ Grade 2 RP in the present study, and 65% of V5 is thus considered to be a threshold value for increased risk of development of ≥ Grade 2 RP. These results have been summarized in Table 3.

**Table 3.** Literature review of developing radiation pneumonitis

Authors	Primary/metastatic	RT technique	Incidence of symptomatic RP	Major contributor	Comments
Graham *et al.* [8]	primary	3DCRT	0–4% (V20 < 25%)	V20	
			19–30% (V20 > 37%)		
Borst *et al.* [11]	primary	SBRT	10.9%	MLD	
		CFRT	17.6%		
Guckenberger *et al.* [12]	primary and metastatic	SBRT	18.6%	V2.5	
Gopal *et al.* [13]	primary	CFRT	60.9% (shortness of breath and exertional dyspnea)	V13	threshold value of deteriorating DLCO
Yamashita *et al.* [14]	primary and metastatic	SBRT	28%	irradiated lung volume-	larger volume of lung being irradiated to high dose
Song *et al.* [15]	primary	HT	18%	V5	≥Grade 3
Present study	metastatic	HT	20.0%	V5	

RT = radiation therapy, RP = radiation pneumonitis, CFRT = conventional fractionated radiation therapy, SBRT = stereotactic body radiotherapy, MLD = mean lung dose, DLCO = diffusion capacity of carbon monoxide, HT = helical tomotherapy.

Our incidence of symptomatic RP was higher than that of previous authors [16], because our patients included some with multiple lung metastases (more than five). As a result, the lung volume receiving not only a higher radiation dose, but also a lower radiation dose was increased. However, most of the symptomatic RP was Grade 2, and the incidence of Grade 3 RP was only 6.6%. This outcome reflects that of another report. McGarry *et al.* reported a 6.4% incidence of Grade 3 RP after SBRT for medically inoperable early lung cancer [17].

The incidence and severity of RP could also be influenced by previous radiotherapy. Among our 45 patients, 5 patients had previously received radiotherapy. Two patients with breast cancer had received postoperative radiotherapy for breast or chest wall after breast conservation surgery or mastectomy (1.8 Gy daily, totalling 50.4 Gy/28 fractions, 4 years ago, and 59.4 Gy/33 fractions, 6 years ago, respectively), and two patients with thymoma had been treated with postoperative radiotherapy to the mediastinum (2 Gy daily, totalling 50 Gy/25 fractions) after thymectomy, however the interval between helical tomotherapy and mediastinal irradiation was > 5 years. One patient with lung cancer had received SBRT on the left upper lung cancer (12 Gy daily, totalling 48 Gy/4 fractions) and was then treated with left upper lobectomy because of progression of the tumor. He received helical tomotherapy for left lower lung tumors, but he died three months after helical tomotherapy from an unknown cause. The pulmonary function of these patients prior to helical tomotherapy was not thought impaired by previous radiotherapy.

Among pulmonary dosimetric parameters, only V5 was association with the development of ≥ Grade 2 RP with statistical significance, and the threshold value of V5 to predict ≥ Grade 2 RP was 65%. An increased incidence of lung toxicity can occur as the distribution of low-dose radiation to the tumor-free normal lung tissue increases. Hence, we have to decrease V5 to < 65% to reduce the risk of symptomatic RP, in addition to considering conventional dosimetric factors. However, limitations of the present study, such as its retrospective nature and the small number of patients using definitive rotational radiotherapy, indicate that further clinical prospective study is required.

## FUNDING

The authors wish to acknowledge the financial support of Catholic Medical Center Research Foundation (5-2011-B0001-00017) made in the program year of 2011.
